# Supplementary material for: Establishment of Translational Luciferase-Based Cancer Models to Evaluate Antitumoral Therapies
Source: Int J Mol Sci. 2024 Sep 27;25(19):10418. doi: 10.3390/ijms251910418 (PMC11476533; doi:10.3390/ijms251910418)
Supplement: Supplementary file 1 [file ijms-25-10418-s001.zip › ijms-3210116-supplementary.pdf]

Supplementary Materials

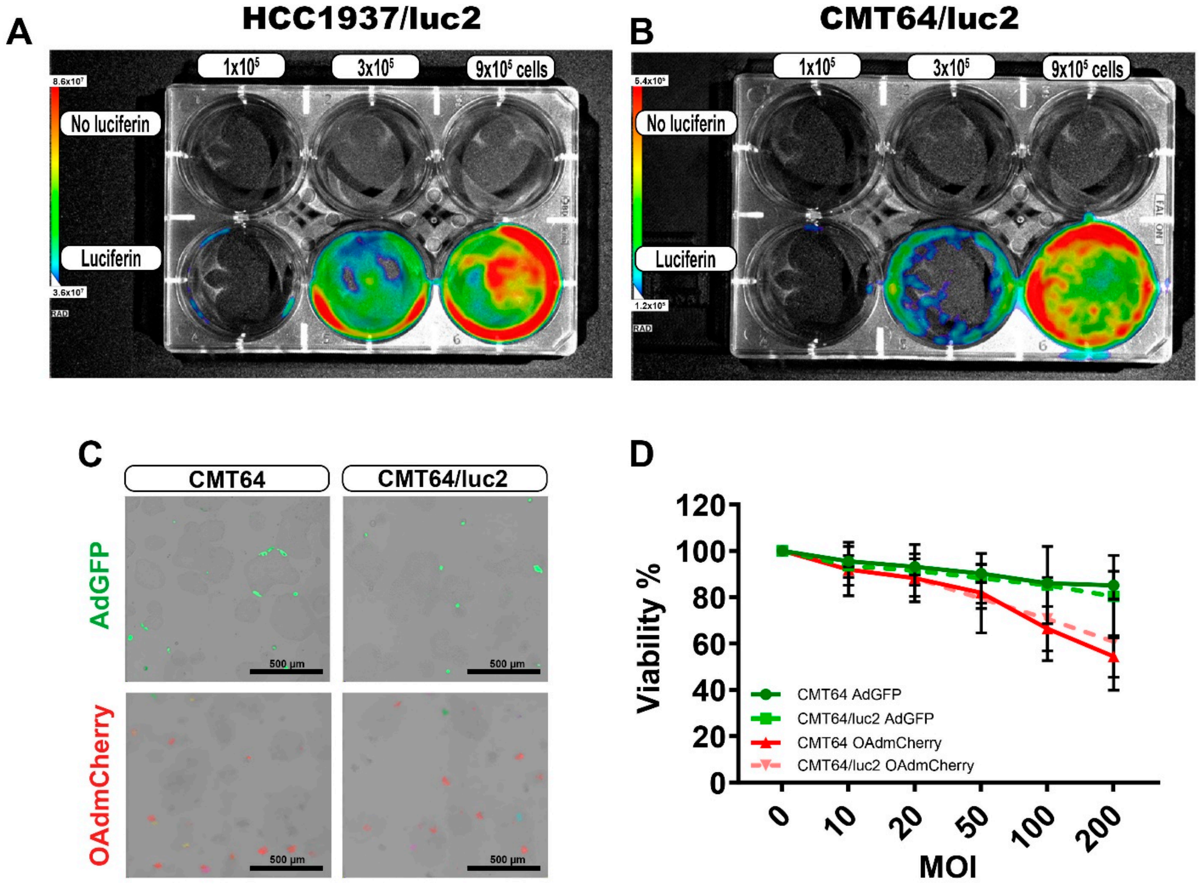

Figure S1. Establishment of the luciferase expressing TNBC (HCC1937/luc2) and lung cancer (CMT64/luc2) cell lines *in vitro*. After incorporating the luciferase plasmid to the parental cell lines, we confirmed the detection of BL signal in both cell lines we modified in our laboratories, the TNBC HCC1937/luc2 (A) and the lung cancer CMT64/luc2 (B). Fluorescent microscopy to evaluate the infectivity capacity using a non-replicative control, AdGFP, and the replication of the OAdmCherry was evaluated in the parental cell line CMT64 and the luciferase modified CMT64/luc2, not showing any significant differences (C). The changes in viability after AdGFP and OAdmCherry was compared between both CMT64 cell lines without any noticeable difference in the killing effect response after 72 h post-infection (D). Graph show 3 independent experiments (Mean ± SD).

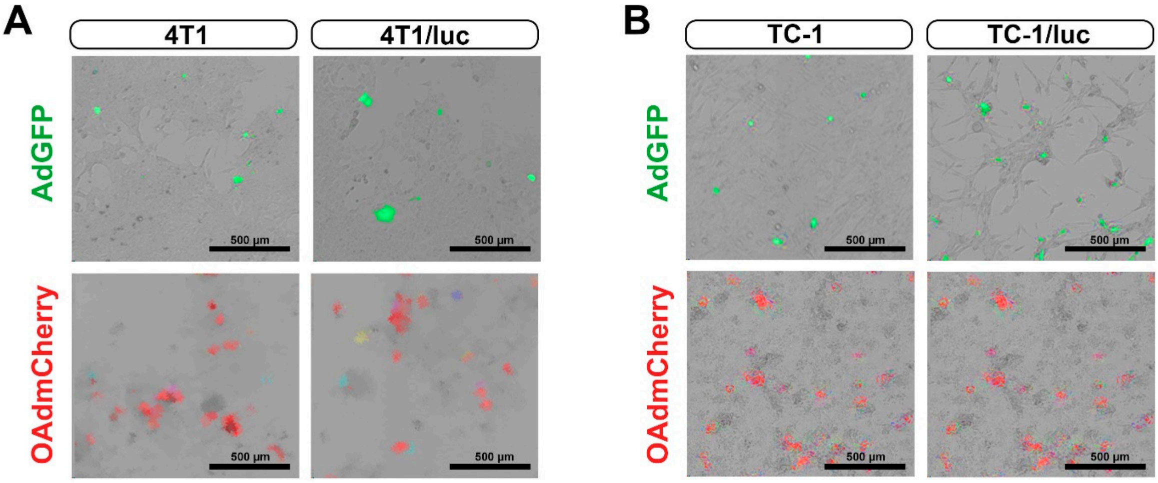

Figure S2. Infectivity and replication sensitivity assay comparing two commercially available cell lines expressing luciferase. We evaluated the production of the encoded fluorescent proteins after treating for 48 h with our infectivity control (AdGFP) and the replication positive control (OAdmCherry) in both, parental and luciferase lung cancer TC-1 cell line (A), as well as in the pair for the TNBC 4T1 cell line (B). There were no differences between the observed infectivity and replication capacities between pairs.
